# Prize Essays and the Medical Association of Georgia

**Published:** 1882-08

**Authors:** 


### PRIZE ESSAYS AND THE MEDICAL ASSOCIATION OF GEORGIA.

At the Thomasville meeting of the State Association in 1881, acting upon the advice of President LeHardy, a committee on Prize Essays was appointed, and was authorized to offer three prizes—one of fifty dollars and two of twenty-five dollars each—to adopt necessary rules governing their bestowment, and to make requisite publication of the offer and of the regulations of the committee in relation thereto.

The committee was judiciously selected, and discharged its duties faithfully and well.

It was hoped by the friends of the Association that this feature of the annual reunions would serve to awaken a renewed interest in the body among the medical men in the state, and to stimulate the members to increased activity in promoting the worthy aims of the organization.

It was discovered, however, when the time for receiving the essays expired, that only one essay was presented, and that solitary claimant was adjudged, by the committee, unworthy of a prize; so that even the most modest anticipations of those who had been most active in the movement were not realized.

Now, it seems to us pertinent to inquire concerning the cause of this total and unhappy failure, with a view, if possible, of averting such an undesirable issue at the next meeting of the Association.

Without presuming to intrude our views upon the committee, it seems to us that the subject selected for the first prize must, almost of necessity, exclude many persons from competing, on account of the absence of facilities enjoyed by residents in cities and towns—the very class of practitioners most likely to enter the lists—for procuring requisite data out of which to construct acceptable and creditable essays.

Then it appears to us that it is a mistake to divide the prize fund into three distinct prizes—the stimulus would be much greater if a single prize of one hundred dollars should be offered, and we predict a larger number of essays under that plan than can be secured under the one adopted last year.

We therefore take the liberty of suggesting to the committee the consolidation of the three prizes, and the selection of a subject—should it be deemed best to name the subject for the essays—that will be within the reach of all, or nearly all, of the physicians of the state, certainly within the reach of that class from whom most effort may reasonably be expected in this direction.

---

OFFICE OF THE DEAN,
SOUTHERN MEDICAL COLLEGE,
ATLANTA, GA., July 15, 1882.

*Dr. James B. Baird, Editor Atlanta Medical Register:*

DEAR DOCTOR:—In the July number, 1882, of the REGISTER, in an editorial entitled "Medical Colleges and Quackery," you have censured justly a medical college for endorsing with its diploma a notorious manufacturer of a secret remedy; but since you failed to mention by name either the school or the graduate, I beg that you will do us the justice to state that the article did not refer to the Southern Medical College. I am yours very truly,

WM. PERRIN NICOLSON, M.D., Dean.

